# Short-Lived Neutralizing Antibody Responses to Monkeypox Virus in Smallpox Vaccine–Naive Persons after JYNNEOS Vaccination

**DOI:** 10.3201/eid3102.241300

**Published:** 2025-02

**Authors:** Kara Phipps, Jennifer Yates, Jessica Pettit, Sean Bialosuknia, Danielle Hunt, Alan P. DuPuis, Anne Payne, William Lee, Kathleen A. McDonough

**Affiliations:** Wadsworth Center, New York State Department of Health, Albany, New York, USA (K. Phipps, J. Yates, J. Pettit, S. Bialosuknia, D. Hunt, A.P. DuPuis II, A. Payne, W. Lee, K.A. McDonough); University at Albany, Albany (J. Yates, K.A. McDonough)

**Keywords:** monkeypox virus, mpox, viruses, orthopoxvirus, smallpox, virus neutralization, plaque reduction neutralization test, PRNT, immune correlates, JYNNEOS, MVA, MVA-BN, vaccine-preventable diseases, vaccines, United States

## Abstract

JYNNEOS, a third-generation smallpox vaccine, is integral to monkeypox virus (MPXV) control efforts, but the durability of this modified vaccinia Ankara–Bavarian Nordic (MVA-BN) vaccine’s effectiveness is undefined. We optimized and used a plaque reduction neutralization test (PRNT) with authentic clade IIa MPXV and vaccinia virus to assess antibody responses over 12 months in 8 donors vaccinated with 2 doses of JYNNEOS. One donor previously received the ACAM2000 vaccine; 7 donors were smallpox vaccine–naive. IgG responses of the donors to vaccinia virus (L1, B5, and A33) or MPXV (E8, H3, A35) antigens and PRNT titers to both viruses peaked at 8 weeks postvaccination and waned rapidly thereafter in naive donors. MPXV PRNT titers were especially low; no naive donors demonstrated 90% plaque reduction. These data indicate a need for improved correlates of MPXV immunity to enable MVA-BN durability studies, given that recent clinical data support MVA-BN vaccine efficacy against MPXV despite low antibody responses.

Monkeypox virus (MPXV), the causative agent of mpox disease, is an ongoing public health concern in the United States and internationally. In 2022, a large global outbreak of mpox spread primarily among men who have sex with men. The 2022 outbreak heightened awareness of the need for preventive measures against transmission and severe mpox disease, triggering a public health campaign that included recommending behavioral changes and vaccination with the modified vaccinia Ankara–Bavarian Nordic (MVA-BN) vaccine JYNNEOS (Bavarian Nordic, https://www.bavarian-nordic) for populations most at risk. MPXV infection is considered endemic in areas of central and western Africa, where it causes thousands of cases annually and where a current multicountry outbreak of clade Ib MPXV infection has escalated to what the World Health Organization has declared a Public Health Emergency of International Concern (*1*,*2*).

MPXV is a member of the *Orthopoxvirus* genus and is related to variola virus, the causative agent of smallpox, and to less virulent genus members, including cowpox virus and vaccinia virus (VACV). Vaccination with VACV provided protection from smallpox and led to its eradication. VACV-based smallpox vaccines are expected to protect against mpox because of cross-reactivity between VACV and MPXV antigens (*3*,*4*). First- and second-generation smallpox vaccines, which consist of replication competent strains of vaccinia, are not recommended for the general population because of potentially severe or fatal side effects for some persons, including those with HIV (*5*). MVA-BN is considered a safer, third-generation smallpox vaccine because it is a highly passaged vaccinia strain; however, unlike prior smallpox vaccines, MVA-BN does not replicate in humans.

Understanding of the protection MVA-BN provides against MPXV is incomplete and emerging. The US Food and Drug Administration approved use of MVA-BN for mpox prevention under the brand name JYNNEOS (Bavarian Nordic) in 2019 (*6*), whereas the European Medicines Agency approved it under the brand name IMVANEX in 2022 (*7*). Epidemiologic studies from the United States support vaccine efficacy for MVA-BN and have estimated its effectiveness against mpox to range from 66% to 88.5% in fully vaccinated persons (*8*–*11*). However, duration for many of those studies was <1 year after the peak of MVA-BN vaccine administration in the United States, so the potential for waning efficacy was not fully captured. Determining the role of MVA-BN in quelling the mpox outbreak in the United States has been challenging because the effects of behavioral changes on mpox transmission are difficult to quantify. Paredes et al. (*12*) modeled infection rates during the 2022 epidemic and concluded that mpox transmission dropped dramatically before vaccination-induced immunity could play a role. Virus-specific neutralizing antibody (nAb) titers are considered an indicator of the smallpox vaccination response and have served as a metric for evaluating noninferiority in clinical studies of MVA-BN (*13*,*14*). We characterized the durability of nAb response generated by the JYNNEOS vaccine to MPXV in a small cohort of naive donors by using a native MPXV plaque reduction neutralization test (PRNT).

## Materials and Methods

### Samples and Ethics 

We conducted this assay development study by using deidentified serum and plasma samples for a public health function in a declared Public Health Emergency. This activity has been deemed non–human subjects research by the New York State Institutional Review Board. The vaccinee cohort consists of serum samples from 8 New York State Department of Health employee donors who were vaccinated with JYNNEOS because of potential occupational exposure.

### Recombinant Orthopoxvirus Antigens

We obtained recombinant proteins from several sources. We obtained recombinant A33 (VAC-WR-A33R), B5 (VAC-WR-B5R), and L1 (VAC-WR-L2R) from BEI Resources. We purchased mpox A35, E8, and H3 from Ray Biotech (https://www.raybiotech.com).

### Orthopoxvirus-Specific Multiplex Microsphere Immunoassay 

We assessed specimens for the presence of antibodies reactive to orthopoxvirus antigens by using a multiplex microsphere immunoassay, as previously described (*15*). We linked recombinant proteins covalently to the surface of fluorescent, magnetic microspheres (Luminex MagPlex Microspheres; Diasorin, https://us.diasorin.com). We mixed serum or plasma samples (25 μL at 1:100 dilution) and antigen-coupled microspheres (25 μL at 5 × 10^4^ microspheres/mL, per manufacturer instructions) and incubated them for 30 minutes at 37°C. We washed serum-bound microspheres and incubated them with phycoerythrin-conjugated secondary antibody specific for human IgG (Southern Biotech, https://www.southernbiotech.com). After washing and final resuspension of samples in buffer, we analyzed them on a FlexMap 3D analyzer (Diasorin) by using xPONENT version 4.3 (Diasorin).

### Calculation of Cutoffs and Index Values

We generated receiving operator characteristic (ROC) curves in GraphPad Prism 9.1.0 (https://www.graphpad.com) for each antigen on the basis of the mean fluorescence intensity (MFI) values of 120 MPXV-negative donors born after 1970 and 40 MPXV-positive confirmed donors, as previously described (*15*). We used sensitivity and specificity values generated by the ROC curve to calculate cutoffs with a Youden J index (J = sensitivity + specificity – 1) for the range of MFI values in the ROC analysis. We set the cutoff value as the MFI equaling the highest Youden J index, which represents the best balance of specificity and sensitivity over the range of the assay. We normalized MFI signals for antigen comparisons for background fluorescence by using an index value (MFI/clinical cutoff).

### Viruses and Cells

We obtained the following reagents through the Biodefense and Emerging Infections Research Resources Repository (BEI Resources, https://www.beiresources.org) at the National Institutes of Health’s National Institute of Allergy and Infectious Diseases: VACV, Western Reserve (National Institute of Allergy and Infectious Diseases, tissue-culture adapted) NR-55; MPXV, USA-2003, NR-2500; and MPXV, Walter Reed Army Institute of Research 7-61, NR-27. We passaged virus stocks once in Vero E6 cells (African green monkey kidney, American Type Culture Collection CRL-1587) maintained in Eagle minimum essential medium (EMEM) with 2% heat-inactivated fetal bovine serum, penicillin (100 unit/mL), and streptomycin (100 μg/mL).

### Sonication

**Figure 1:**
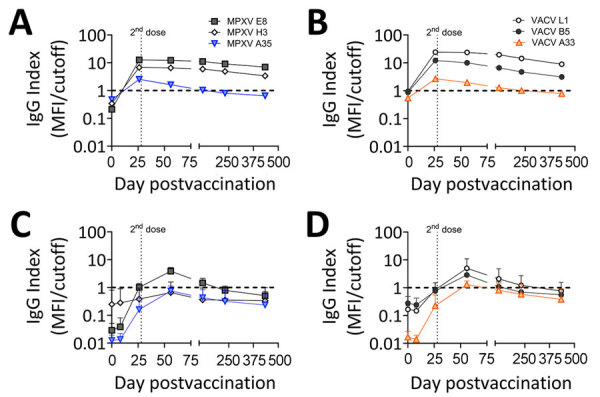
IgG reactivity to orthopoxvirus antigens in JYNNEOS vaccinees with and without prior smallpox vaccination in a study to assess neutralizing antibody responses to MPXV in smallpox vaccine–naive persons after JYNNEOS vaccination. We analyzed serum specimens from 8 JYNNEOS vaccine recipients for IgG reactivity to recombinant protein antigens derived from MPXV or VACV by using multiplex microsphere immunoassay. One donor who received ACAM2000 vaccine before JYNNEOS vaccine is shown separately in panels A and B. Means of 7 persons who had no prior smallpox vaccination are shown in panels C and D. We plotted mean index values (MFI/cutoff) of MPXV E8 (gray squares), MPXV A35 (blue triangles), and MPXV H3 (white diamonds) for days 0, 8, 26, 56, 118, 231, and 434 postvaccination (panels A, C). We plotted mean index values (MFI/cutoff) of VACV L1 (white circles), VACV A33 (orange triangles), and VACV B5 (gray circles) for days 0, 8, 26, 56, 118, 231, and 434 postvaccination (panels B, D). The horizontal black dashed line at y = 1.0 indicates the cutoff value. The vertical dotted line indicates the second dose of vaccine at day 28 postvaccination. MFI, mean fluorescence intensity; MPXV, monkeypox virus; VACV, vaccinia virus.

**Figure 2:**
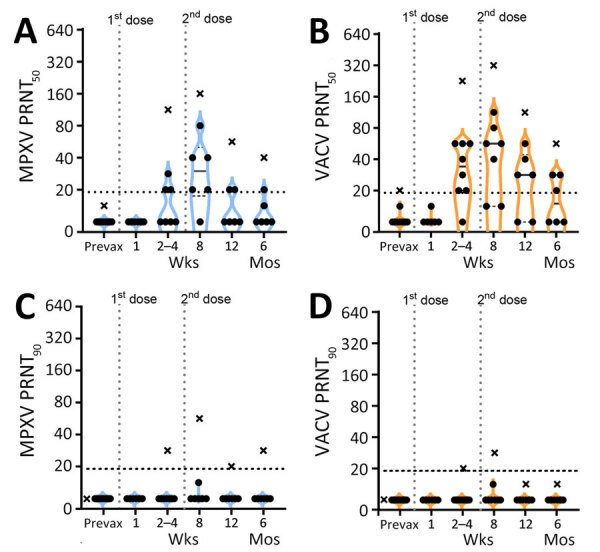
PRNT titers for participants vaccinated with JYNNEOS vaccine up to 6 months prior in a study to assess neutralizing antibody responses to MPXV in smallpox vaccine–naive persons after JYNNEOS vaccination. We used PRNT to test serum samples from donors vaccinated with 2 doses of JYNNEOS vaccine ≈28 days apart. We performed assays with sonicated virus and a 1-hour virus–serum incubation. A) MPXV PRNT_50_ results; B) VACV PRNT_50_ results; C) MPXV PRNT_90_ results; D) VACV PRNT_90_ results. Participants with no known vaccinia exposure (black circles) are used for mean calculations. Data from a single donor with prior smallpox vaccination (black Xs) are plotted separately and excluded from mean calculations. Each datapoint represents the geometric mean titer of 2 independent experiments. The vertical dotted lines represent the timing of the vaccine doses, and the horizontal dotted lines indicate limits of detection. MPXV, monkeypox virus; prevax, prevaccination; PRNT, plaque reduction neutralization test; PRNT_50_, 50% plaque reduction as measured by PRNT; PRNT_90_, 90% plaque reduction as measured by PRNT; VACV, vaccinia virus.

We performed sonication in sealed tubes with the Virtis Virsonic 100-cup horn sonicator continuously cooled to 4°C with a circulating water bath. We diluted virus in EMEM with 2% heat-inactivated fetal bovine serum, and we sonicated separate aliquots with increasing intensity at settings 2, 3, 4, and 5 for four 5-second bursts separated by 5-second rest intervals to determine optimal sonication conditions (Appendix Table 1, Figures 1, 2). Thereafter, we used intensity setting 3 as part of a standardized protocol.

### PRNT

Virus strains used in PRNT were VACV Western Reserve and MPXV USA-2003. We did not heat-inactivate test serum unless otherwise noted. We serially diluted each serum sample 2-fold in EMEM with 2% heat-inactivated fetal bovine serum. We sonicated an equal volume of media containing either VACV or MPXV at setting 3 and added it to each sample at a concentration expected to yield ≈100 PFU. We incubated virus–serum mixtures at 37°C for 1 hour, with the exception of experiments that lasted 24 hours (Appendix Figure 3). We then inoculated the mixture onto Vero E6 cell monolayers and adsorbed them for 1 hour at 37°C. We added EMEM media containing 0.6% oxoid agarose to wells, allowed them to solidify, and incubated them at 37°C with 5% CO_2_. We added a secondary overlay containing 0.2% neutral red (Sigma-Aldrich, https://www.sigmaaldrich.com) for plaque visualization at 48 hours postinfection. We determined the timing of the secondary overlay after finding that overlays performed at 48 hours and 72 hours produced similar results (Appendix Table 2). We counted plaques 24 hours after the second overlay. We determined neutralization titers to be the serum dilution resulting in a 50% (PRNT_50_) or 90% (PRNT_90_) plaque reduction compared with the virus working dilution (≈100–250 PFU). We incubated virus inoculum and used it to enumerate the working dilution in media alone alongside samples containing virus–serum before infection and then titrated it by using a plaque assay in parallel to PRNT. We included positive- and negative-control antibodies in each assay, and we rejected assay results with a 4-fold difference in the range of control antibodies. PRNT titers measuring the efficacy of JYNNEOS in vaccinated donors over time are the result of 2 independent experiments, except for the experiments using only a single assay (Appendix Figure 3). We gave samples that did not neutralize at the 1:20 limit of detection an arbitrary neutralization value of 1:10 for geometric mean titer (GMT) calculations.

### Statistical Analyses

We used 1-way analysis of variance to assess statistical significance. For multiple comparisons of the differences in means of >3 groups to a control group, we used 1-way analysis of variance followed by Dunnett multiple comparison test.

## Results

We performed studies with donated serum samples from persons (n = 8) vaccinated with a 2-dose regimen of the JYNNEOS vaccine against potential occupational exposure. Vaccine doses were administered ≈28 days apart, and serum samples were collected from all participants shortly before JYNNEOS vaccination and at sequential time points until 12 months postvaccination. Seven donors were administered the vaccine subcutaneously, and 1 donor received the vaccine intradermally. One donor had received ACAM2000 (Sanofi, https://www.sanofi.com), a second-generation smallpox vaccine, ≈5 years before JYNNEOS vaccination. The remaining donors were determined to be previously smallpox vaccine–naive on the basis of their personal account, a lack of a vaccine take scar, their age, or a combination of those factors. Because of differences in timing of vaccination, the 12-month sampling point included serum samples for only 7 of the 8 participants.

We examined donor serum samples for IgG reactivity to MPXV- and VACV-derived antigens by using a previously described microsphere immunoassay (*15*) to assess overall antibody levels and cross-reactivity to MPXV in response to JYNNEOS vaccination. Orthopoxvirus virions have 2 forms, which differ in their surface proteins, intracellular mature virions (IMVs) and extracellular enveloped virions, so we tested antigens from each form. VACV L1 and MPXV E8 and H3 antigens are found on IMVs, whereas the remaining antigens are found on extracellular enveloped virions. We selected VACV recombinant proteins L1, A33, and B5 for quantification because immunization by those antigens and VACV A27 demonstrated protection from lethal mpox in nonhuman primates (*16*). We selected MPXV recombinant protein antigens on the basis of commercial availability.

Serum samples from the donor with prior smallpox vaccination (Figure 1, panels A, B) displayed much higher IgG reactivity than did the samples from the naive donors, so we excluded those samples from the mean values (Figure 1, panels C, D). The previously vaccinated donor produced detectable IgG response for all MPXV and VACV antigens. In naive donors, the mean serum IgG reactivity became positive for all VACV antigens assayed; VACV L1 showed the highest mean IgG reactivity of all antigens tested (Figure 1, panel D). E8 was the sole MPXV antigen with positive mean IgG reactivity in naive donors, despite MPXV A35 being homologous to VACV A33 (Figure 1, panel C). We also noted that serum samples from all donors reacted most strongly to IMV antigens from both viruses (L1 and E8). For all antigens, IgG reactivity peaked at ≈8 weeks after the initial dose and waned thereafter, indicating the antibody response generated by JYNNEOS is short-lived in naive persons (*17*). Serum samples from the previously vaccinated person remained stably positive beyond 250 days postvaccination for all antigens except MPXV A35 (Figure 1, panels A, B).

PRNT is considered the standard for measuring nAb levels because it directly measures inhibition of native virus infection. We developed our PRNT by making minor modifications to a standard assay (*18*,*19*). Orthopoxviruses such as VACV are known to form multivirion aggregates (*20*,*21*), and such structures can affect antibody-binding interactions and neutralizing properties (*22*,*23*). Sonication has been used with VACV infections, but more recent MPXV studies have omitted this step either in practice or in reporting (*24*–*29*). Because preliminary MPXV assays showed variability and nonuniform plaque clusters (Appendix Figures 1, 2), we introduced a sonication step. We empirically determined the sonication conditions of VACV and MPXV stocks used in our study by sonicating at increasing levels of intensity with a cup horn sonicator. Although plaque titrations of MPXV without sonication produced visible clusters of plaques that prevented accurate titer estimation, low levels of sonication treatment resulted in well-separated MPXV plaques and significantly increased titers (p = 0.0166) (Appendix Figure 2). We selected intensity setting 3 for subsequent use because it was the lowest setting that provided significantly increased plaque numbers for both viruses (p = 0.0185) (Appendix Figure 2). We sonicated virus by using this procedure at the start of each PRNT.

We also considered the duration of virus incubation with serum samples before infection, given that some PRNT studies of MPXV and VACV neutralization extend the virus–serum incubation to overnight rather than 1 hour at 37°C (*24*,*30*). We found that the extended incubation time was suboptimal despite producing increased PRNT_50_ titers because infectivity of the viruses also decreased independently of nAb with the extended adsorption time. MPXV demonstrated a 43.2% reduction in mean working dilution (p<0.00001), whereas VACV demonstrated a 20.9% reduction (p = 0.00121) (Appendix Figure 3, panel B). This decreased infectivity suggested virus instability during the extended incubation time, which was greater for MPXV than VACV (Appendix Figure 3, panel B).

**Figure 3:**
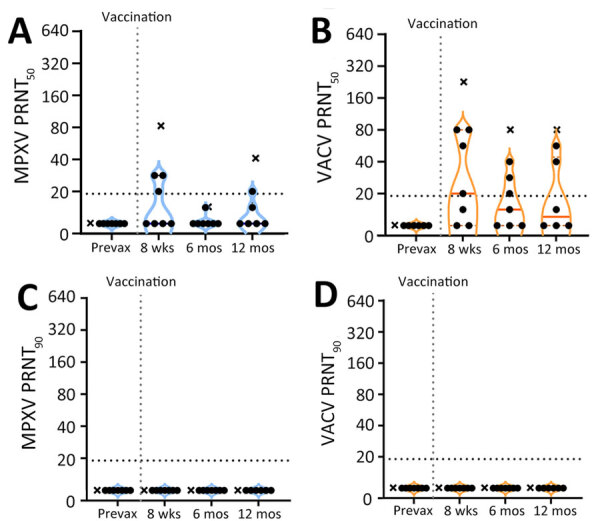
Longitudinal neutralizing antibody response by JYNNEOS vaccination extending to 12 months in a study to assess neutralizing antibody responses to MPXV in smallpox vaccine–naive persons after JYNNEOS vaccination. We used PRNT to test serum samples from donors vaccinated with 2 doses of JYNNEOS ≈28 days apart. We performed assays with sonicated virus and a 1-hour virus–serum incubation. A) MPXV PRNT_50_ results; B) VACV PRNT_50_ results; C) MPXV PRNT_90_ results; D) VACV PRNT_90_ results. Data from a single donor with prior smallpox vaccination are plotted separately (black Xs). We used data from participants with no known vaccinia exposure for mean calculations (black circles). Each datapoint represents the geometric mean titer of 2 independent experiments, and the limits of detection are expressed by horizontal dotted lines. MPXV, monkeypox virus; prevax, prevaccination; PRNT, plaque reduction neutralization test; PRNT_50_, 50% plaque reduction as measured by PRNT; PRNT_90_, 90% plaque reduction as measured by PRNT; VACV, vaccinia virus.

We used PRNT to measure nAb responses for MPXV and VACV (Figure 2). Samples from the donor with prior smallpox vaccination had higher levels of neutralization than did the samples from naive donors (Figures 2, 3). The previously vaccinated donor was also the only person whose sample produced a positive PRNT_90_ result (Figure 2). Because of the difference in vaccination history, datapoints from that person are shown in plots (Figures 2, 3) but were excluded from the overall mean PRNT titer calculations.

In all previously naive persons tested, nAb responses toward MPXV peaked at an average geometric mean PRNT_50_ titer (GMT) of 1:35 ≈1 month after the second dose of JYNNEOS and quickly waned to below the 1:20 limit of detection (LOD) (Figure 2). Neutralization of VACV was better than MPXV after only 1 dose of vaccine and was more robust, having a peak GMT PRNT_50_ of 1:61 at 8 weeks after the initial dose (Figures 2, 3). Neutralization of either virus waned similarly over time after vaccination (Figure 2). One person mounted no detectable neutralization response to MPXV (Figure 2, panel A). For most persons, PRNT_50_ titers to MPXV and VACV were below the LOD for both viruses by 12 weeks after the initial vaccine dose (Figure 2, panels A, B). At 12 months, postvaccination serum samples from previously naive persons retained some reactivity to VACV (PRNT_50_ GMT 1:23), but neutralization of MPXV was at or below the PRNT_50_ LOD (GMT 1:12) (Figure 3). No naive donors produced a detectable PRNT_90_ titer of >1:20 to either MPXV or VACV at any timepoint.

## Discussion

The low levels of MPXV-neutralizing activity induced by JYNNEOS vaccination observed in this study are consistent with results of other recent studies, some of which have raised concerns over the efficacy and durability of MVA-BN vaccines in preventing mpox disease and spread (*26*–*29*,*31*). We also found that neutralization titers can be affected by assay conditions, which should be considered when comparing neutralizing activity levels from different studies. Empirical testing of MPXV PRNT assay conditions showed that sonication improves MPXV plaque quality and assay reliability, supporting its inclusion as part of a standardized protocol, as it is for VACV (*32*). In addition, significantly reduced MPXV infectivity with an overnight preincubation period (Appendix Figure 3, panel B) leads us to propose that shorter preincubation times are preferable for MPXV neutralization assays, despite some increased sensitivity with extended preincubation.

One limitation of this study is the small number of donors that were assessed. Nonetheless, our results align well with those of studies that had greater numbers of participants (*27*,*28*,*31*). In addition, our antigen-binding data are limited by the subset of MPXV and VACV antigens that were measured. Protective epitopes from MPXV have not been well defined and might not have been present among the tested antigens.

The low nAb levels that we and others (*27*–*29*,*31*) observed after MVA-BN vaccination of smallpox vaccine–naive persons differ from those of prior studies that showed first-generation smallpox vaccines can produce a long-lasting humoral response to MPXV (*25*,*29*,*33*). This durable antibody response is consistent with the finding that nAb levels remain elevated for decades in those who recover from smallpox infection, a condition that is assumed to produce lifelong immunity (*34*). In addition, a previous study comparing the effect of depleting either B cell or CD8 T-cell responses in a nonhuman primate challenge model led to the conclusion that nAb responses are a primary means of protection against mpox (*35*). Animals immunized with a first-generation smallpox vaccine demonstrated that an intact humoral response alone or passive antibody transfer was sufficient to protect the animals from lethal MPXV infection (*35*). Despite our observation of a limited antibody response after JYNNEOS vaccination, recent reports indicate that MVA-BN vaccine efficacy against mpox is strong (*36*,*37*) and breakthrough infections that occur in a minority of vaccinated persons (*10*) generally result in mild disease (*38*–*40*). In contrast to studies with earlier-generation smallpox vaccines, these results suggest that sustained nAb levels are not the most reliable correlate of immunity to mpox after MVA-BN vaccination, which is a critical issue for further investigation.

ACAM2000 is a closely related derivative of first-generation smallpox vaccines that are known to produce durable antibody responses (*33*). However, long-term studies on the durability of the antibody response generated by ACAM2000 against either VACV or MPXV in humans were not available when the US Food and Drug Administration established noninferiority of JYNNEOS to ACAM2000 (*13*,*14*,*41*). Because of the difference in its replicative ability postvaccination, the durability and the specificity of immune response elicited by MVA-BN to MPXV might bear less similarity to historical smallpox vaccination than expected and should continue to be evaluated.

The donor who received prior ACAM2000 vaccination produced a greater IgG response and higher neutralization titers than naive donors. However, the extent to which that person’s nAb response to MPXV was affected by intrinsic differences between ACAM2000 and JYNNEOS (e.g., replication competence) versus the boosting of a memory response by additional vaccine doses is unclear and warrants further investigation. Other studies suggest that nAb responses to MPXV can be enhanced by either a third MVA-BN dose after the initial MVA-BN 2-dose series or MVA-BN vaccination after a first-generation smallpox vaccination (*25*,*29*). Both immunization strategies produced elevated nAb levels to VACV that were stable when measured out to 6 months (*42*). MPXV nAb levels can likewise be enhanced by either strategy, albeit to a lesser degree (*25*,*28*,*29*). Two doses of MVA-BN after historical smallpox vaccination can boost MPXV nAb levels out to 1 year (*28*). A study that measured MPXV nAb levels after a third dose of a recombinant modified vaccina Ankara engineered to express influenza H5 protein did so only up to 4 weeks after dose 3 (*29*), and further study is needed to address the durability of this boosted response to MPXV in naive persons.

It is possible that protection against mpox after MVA-BN vaccination is more dependent on memory B cells, production of a robust cellular immune response, or both, compared with the earlier-generation smallpox vaccines. Rhesus macaques vaccinated with recombinant MVA containing HIV or simian human immunodeficiency virus genes survived a lethal dose of MPXV up to 3 years postvaccination, despite most animals displaying low nAb levels before MPXV challenge (*43*,*44*). Cohn et al. (*17*) found that JYNNEOS vaccination led to an increase in CD4 and CD8 T cells that could recognize and respond to orthopoxvirus-specific antigens. Those CD4 and CD8 T-cell responses from the JYNNEOS 2-dose recipients were similar to those of MPXV-convalescent donors. Cytokine responses were also comparable in the vaccinated versus convalescent groups. A comparative challenge study of rhesus macaques immunized with MVA-BN or ACAM2000 also found that both vaccines produced similar T-cell responses to VACV lysate (*45*). Furthermore, T-cell responses to MVA-BN antigens were found to persist in a group of MVA-BN vaccinees when tested out to 1 year (*28*).

Further examination of immune durability and correlates of protection in MVA-BN vaccinees is urgently needed to address public health concerns associated with the ongoing spread of mpox. Topics of particular importance include the roles of memory B-cell and T-cell responses in mpox immunity and the immune mechanisms engendered by earlier-generation replication-competent smallpox vaccines versus MVA-BN. Understanding the mechanisms by which the third-generation MVA-BN vaccine generates immunity against MPXV infection will be central to informing public health responses to mpox disease.

Appendix Additional information regarding short-lived neutralizing antibody responses to monkeypox virus in smallpox vaccine–naive persons after JYNNEOS vaccination.
