# Supplementary material for: Short-Lived Neutralizing Antibody Responses to Monkeypox Virus in Smallpox Vaccine–Naive Persons after JYNNEOS Vaccination
Source: Emerg Infect Dis. 2025 Feb;31(2):237–45. doi: 10.3201/eid3102.241300 (PMC11845161; doi:10.3201/eid3102.241300)
Supplement: Appendix — Additional information regarding short-lived neutralizing antibody responses to monkeypox virus in smallpox vaccine–naive persons after JYNNEOS vaccination. [file 24-1300-Techapp-s1.pdf]

Article DOI: https://doi.org/10.3201/eid3102.241300

EID cannot ensure accessibility for supplementary materials supplied by authors. Readers who have difficulty accessing supplementary content should contact the authors for assistance.

Short-Lived Neutralizing Antibody Responses to Monkeypox Virus in Smallpox Vaccine–Naive Persons after JYNNEOS Vaccination

Appendix

Appendix Table 1. Effect of sonication on number of plaque forming units

		Monkeypox virus, USA-2003	Vaccinia virus, Western Reserve
Treatment[a]	Sonicator power setting	Mean fold change of sonicated versus nonsonicated condition	Mean fold change of sonicated versus nonsonicated condition[b]
S2	2	5.22 (3.1–7.68)	4.72 (4.50–5.16)
S3	3	9.59 (5.28–15.19)	4.81 (3.64–6.93)
S4	4	9.82 (8.44–11.81)	5.74 (4.57–7.45)
S5	5	8.19 (5.28–11.72)	4.13 (2.64–6.05)

[a]Monkeypox virus (MPXV) and vaccinia virus (VACV) virus aliquots were sonicated at intensity settings 2, 3, 4, or 5 and subsequently titrated by plaque assay.
[b]The fold change of each biologic replicate was determined by dividing the virus titer of sonication treated aliquots by the non-sonicated aliquot titer. The mean fold change is the result of three biologic replicates.

Appendix Table 2. Effect of incubation period before overlay stain on MPXV plaque titration

	48 h post infection	72 h post infection
Secondary overlay timing	Mean titer (PFU/mL), range (low-high)	Mean titer (PFU/mL), range (low-high)
Experiment 1[a]		
Vaccinia virus, Western Reserve	5.25×10^6 (3.80×10^6–6.70×10^6)	3.95×10^6 (3.50×10^6–4.40×10^6)
Monkeypox virus, WRAIR	4.45×10^5 (4.10×10^5–4.80×10^5)	4.55×10^5 (4.50×10^5–4.60×10^5)
Monkeypox virus, USA-2003	1.15×10^6 (1.11×10^6–1.18×10^6)	1.35×10^6 (1.23×10^6–1.46×10^6)
Experiment 2[b]		
Monkeypox virus, USA-2003	3.73×10^7 (2.60×10^7–4.80×10^7)	3.13×10^7 (2.50×10^7–3.80×10^7)

Plaque titrations were performed for the listed virus strains with secondary overlay being added at either 48 h or 72 h post infection, as described in methods.
[a]Experiment 1 mean titers were calculated by averaging duplicates. No sonication was performed.
[b]Experiment 2 mean titers were calculated by averaging three replicates, each performed in duplicate. Sonication was performed at setting 3.

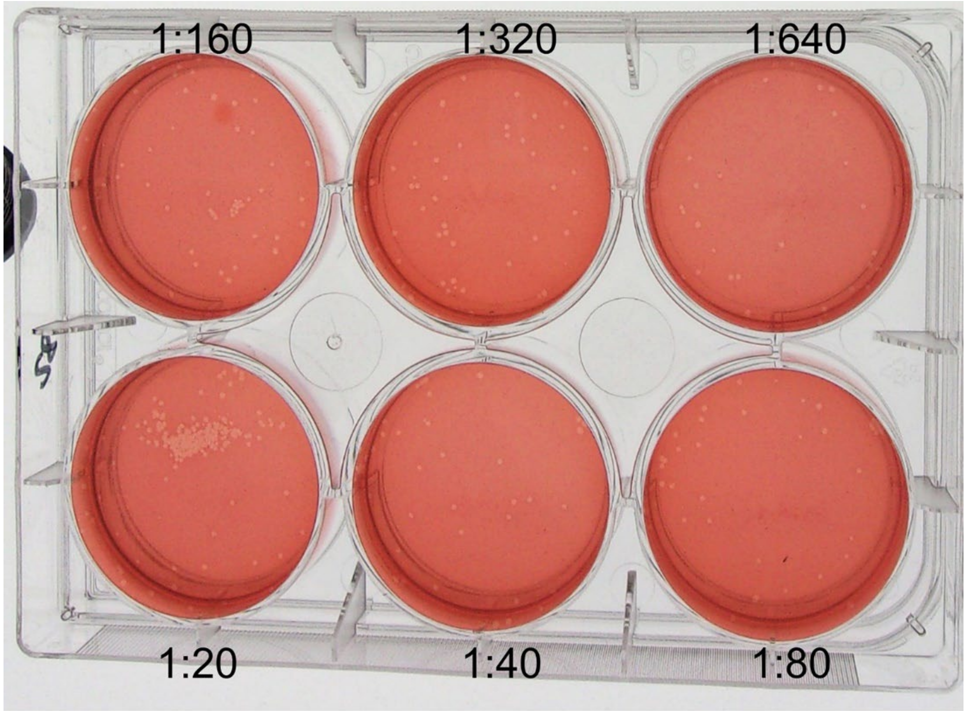

Appendix Figure 1. Plaque clusters in MPXV titration well. Plaques shown for multiple serum dilutions (1:20 through 1:640) in a PRNT of MPXV performed without sonicating virus before inoculation were non-uniform, producing a large plaque cluster in bottom left (1:20) well and small clusters in top left (1:160) well. Image brightness, contrast, and saturation were optimized for visualization.

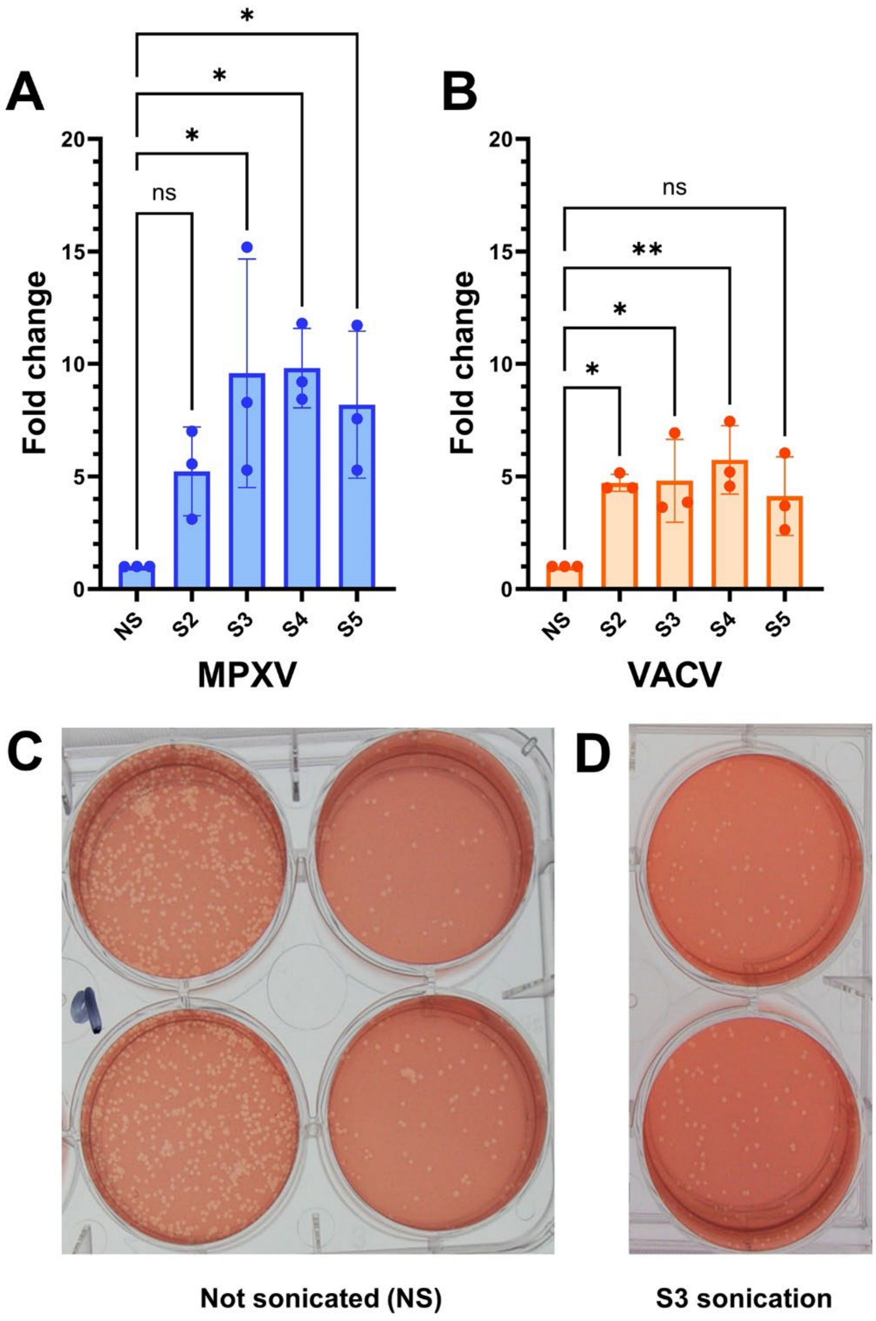

Appendix Figure 2. Optimization of MPXV and VACV sonication. Fold change in virus titer normalized to non-sonicated virus titer for A) MPXV and B) VACV. The mean represents the average of three biologic replicates as in Appendix Table 1. C) Plaque assays of MPXV showing plaque morphology when not sonicated and D) with sonication treatment at setting 3. One-way analysis of variance (ANOVA) with multiple comparisons was used to evaluate significance relative to non-sonicated condition. *, $p<0.05$; **, $p<0.005$. Image brightness, contrast, and saturation were optimized for visualization.

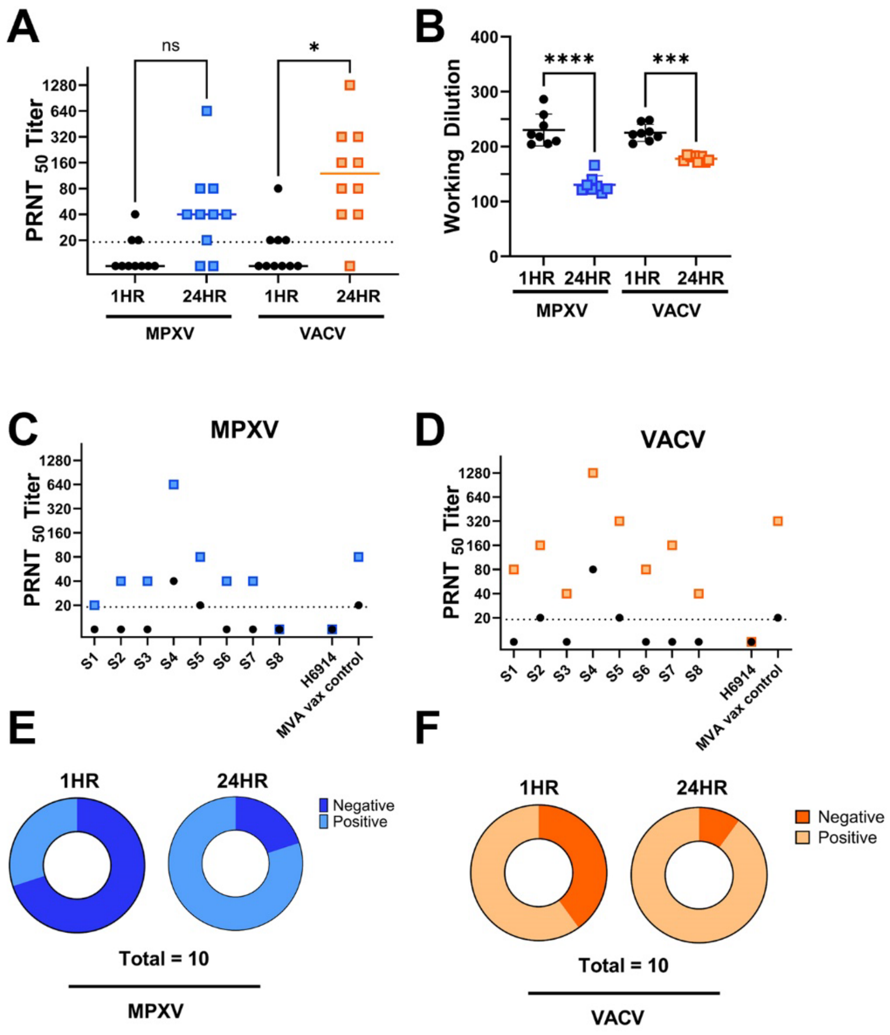

Appendix Figure 3. Evaluation of extended virus–sera adsorption period. Eight independent serum samples of various $PRNT_{50}$ titers were selected and positive and negative controls were tested in parallel by performing a PRNT with a virus–sera incubation step of either one or twenty four hours at 37°C. The negative control consisted of pooled serum product #H61914–20ML Human serum type AB (H6914). The "MVA vax control" used was a donor sample of which the PRNT range had been previously determined through at least three independent assays. $PRNT_{50}$ titers A) Combined $PRNT_{50}$ values for MPXV or VACV B) Working dilution was determined by back titration of virus inoculum in media alone following one and twenty-four hours incubation alongside sera containing samples. After twenty-four hours incubation, MPXV and VACV inoculum titers were reduced by 43.2% and 20.9% respectively as compared to the titer of the same virus preparation when incubated for one hour. Individual data from the summary in A) are shown in C) for MPXV and D) for VACV. $PRNT_{50}$ titers at or above 1:20 are considered seropositive by the limit of detection of our assay and are displayed in lighter colors of E) for MPXV and F) for VACV. One-way analysis of variance (ANOVA) with multiple comparisons was used to compare 1HR and 24HR conditions. *, p<0.05; *, p<0.05; ** P, <0.005; ***, p<0.0005; ****, p<0.00005.
